# Association between Clean Delivery Kit Use, Clean Delivery Practices, and Neonatal Survival: Pooled Analysis of Data from Three Sites in South Asia

**DOI:** 10.1371/journal.pmed.1001180

**Published:** 2012-02-28

**Authors:** Nadine Seward, David Osrin, Leah Li, Anthony Costello, Anni-Maria Pulkki-Brännström, Tanja A. J. Houweling, Joanna Morrison, Nirmala Nair, Prasanta Tripathy, Kishwar Azad, Dharma Manandhar, Audrey Prost

**Affiliations:** 1 UCL, Centre for International Health and Development, Institute of Child Health, United Kingdom; 2 UCL, Centre for Paediatric Epidemiology and Biostatistics, Institute of Child Health, United Kingdom; 3 Ekjut, Chakradharpur, Jharkhand, India; 4 Perinatal Care Project (PCP), Bangladesh; 5 Mother and Infant Research Activities (MIRA), Nepal; Johns Hopkins Bloomberg School of Public Health, United States of America

## Abstract

**Background:**

Sepsis accounts for up to 15% of an estimated 3.3 million annual neonatal deaths globally. We used data collected from the control arms of three previously conducted cluster-randomised controlled trials in rural Bangladesh, India, and Nepal to examine the association between clean delivery kit use or clean delivery practices and neonatal mortality among home births.

**Methods and Findings:**

Hierarchical, logistic regression models were used to explore the association between neonatal mortality and clean delivery kit use or clean delivery practices in 19,754 home births, controlling for confounders common to all study sites. We tested the association between kit use and neonatal mortality using a pooled dataset from all three sites and separately for each site. We then examined the association between individual clean delivery practices addressed in the contents of the kit (boiled blade and thread, plastic sheet, gloves, hand washing, and appropriate cord care) and neonatal mortality. Finally, we examined the combined association between mortality and four specific clean delivery practices (boiled blade and thread, hand washing, and plastic sheet). Using the pooled dataset, we found that kit use was associated with a relative reduction in neonatal mortality (adjusted odds ratio 0.52, 95% CI 0.39–0.68). While use of a clean delivery kit was not always accompanied by clean delivery practices, using a plastic sheet during delivery, a boiled blade to cut the cord, a boiled thread to tie the cord, and antiseptic to clean the umbilicus were each significantly associated with relative reductions in mortality, independently of kit use. Each additional clean delivery practice used was associated with a 16% relative reduction in neonatal mortality (odds ratio 0.84, 95% CI 0.77–0.92).

**Conclusions:**

The appropriate use of a clean delivery kit or clean delivery practices is associated with relative reductions in neonatal mortality among home births in underserved, rural populations.

*Please see later in the article for the Editors' Summary*

## Abstract 2

A pooled analysis of data from three studies in South Asia demonstrates an association between use of clean delivery kits during home births and reduced risk of neonatal mortality.

## Editors' Summary

**Background:**

Worldwide, around 3.3 million babies die in the first month of life, according to data for 2009 from the World Health Organization. Although the global neonatal mortality rate declined by 28% (from 33.2 deaths per 1,000 live births to 23.9) between 1990 and 2009, the proportion of child deaths that are now in the neonatal period has increased in all regions of the world, and currently stands at 41%. This figure is concerning and neonatal mortality remains a big obstacle to the international community in meeting the target of Millennium Development Goal 4—to reduce deaths in children under 5 years by two-thirds from 1990 levels by 2015. At least 15% of all neonatal deaths are due to sepsis (systematic bacterial infection) and an estimated 30%–40% of infections are transmitted at the time of birth. Therefore preventing infections through clean delivery practices is an important strategy to reduce sepsis-related deaths in newborns and can contribute to reducing the overall burden of neonatal deaths.

**Why Was This Study Done?:**

In South Asia, around 65% of deliveries occur at home, without skilled birth attendants, making practices around clean delivery particularly challenging. To date, evidence on the impact of clean delivery kits and clean delivery practices on neonatal mortality or sepsis-related neonatal deaths from community-based studies is scarce. In this study the researchers explored the associations between neonatal mortality, the use of clean delivery kits, and individual clean delivery practices by using data from the control arms of three cluster-randomized controlled trials conducted among rural populations in South Asia.

**What Did the Researchers Do and Find?:**

The researchers used data from almost 20,000 (19,754) home births available from the control arms of three community-based cluster-randomized trials conducted between 2000 and 2008 in India (*n* = 6,841, 18 clusters), Bangladesh (*n* = 7,041, five clusters), and Nepal (*n* = 5,872, five clusters). The researchers did not include data from other previously conducted trials on clean delivery practices because of the mix of designs used in these studies and limited their analysis to live-born singleton infants delivered at home in control areas, for whom data on birth kit use were available. The researchers conducted a separate analysis for each country on kit use and clean delivery practices and also analyzed the pooled dataset for all countries while controlling for factors about the mother, the pregnancy, the delivery, and the postnatal period.

Using these methods, the researchers found that kits were used for 18.4% of home births in India, 18.4% in Bangladesh, and 5.7% in Nepal. Importantly, according to the pooled analysis, kit use was associated with a 48% relative reduction in neonatal mortality (odds ratio/chance 0.52), which was similar across all countries: 57% relative reduction in neonatal mortality in India, 32% in Bangladesh, and 49% in Nepal. Delivery practices were also important: in the pooled country analysis, the use of a boiled blade to cut the cord, antiseptic to clean the cord, a boiled thread to tie the cord, and a plastic sheet for a clean delivery surface were all associated with significant relative reductions in mortality after controlling for kit use and confounders common to all sites. The researchers found a 16% relative reduction in mortality with each additional clean delivery practice used.

**What Do These Findings Mean?:**

These findings show that the appropriate use of a clean delivery kit and clean delivery practices could lead to substantial reductions in neonatal mortality among home births in poor rural communities with limited access to health care. The results also reinforce the importance of each clean delivery practice; hand washing and use of a sterilised blade, boiled thread, and plastic sheet were linearly associated with a reduction in neonatal deaths with each additional clean delivery practice used. Costs of such kits are low (US$0.44 in India, US$0.40 in Nepal, and US$0.27 in Bangladesh, although these costs may still be prohibitive for the poorest women), and given the impact of clean delivery kits and clean delivery practices in reducing neonatal practices, such strategies should be widely promoted by the international community.

**Additional Information:**

Please access these websites via the online version of this summary at http://dx.doi.org/10.1371/journal.pmed.1001180.

- A recent *PLoS Medicine* study by Oestergaard et al. has the latest figures on neonatal mortality worldwide
- UNICEF has information about progress toward Millennium Development Goal 4
- The United Nations Population Fund has more information about safe birth practices
- The EquiNam web site describes ongoing work on socioeconomic inequalities in newborn and maternal health in Asia and Africa by some of the study authors

## Introduction

Every year, an estimated 3.3 million newborn infants worldwide die in the first month of life, 99% of them in low- and middle-income countries, and 35% of them in South Asia [1]–[4]. The fourth Millennium Development Goal set a target to reduce mortality in children by two-thirds between 1990 and 2015 [5]. Although neonatal mortality rates declined by 31% in South Asia between 1990 and 2009, they remain high in many countries: 34.3 (27.7–40.8) per 1,000 live births in India, 31.3 (25.4–36.9) in Bangladesh, and 25.4 (20.5–30.9) in Nepal [3],[4].

Direct cause-of-death data suggest that sepsis, defined as a systemic bacterial infection, could be responsible for up to 15% of neonatal deaths [1]. An estimated 30%–40% of infections leading to neonatal sepsis are transmitted at the time of birth, and early-onset sepsis can manifest within the first 72 h of life [6]. Preventing infections through clean delivery practices is an important strategy to reduce sepsis-related deaths [7]. The World Health Organization (WHO) promotes the observance of “six cleans” at the time of delivery: clean hands, clean perineum, clean delivery surface, clean cord and tying instruments, and clean cutting surfaces [7]. A recent expert consensus suggested that uptake of these practices could reduce neonatal sepsis deaths by 15% for home births (interquartile range [IQR] 10–20) and 27% for facility births (IQR 24–36) [8].

In South Asia, around 65% of deliveries occur at home, most (59%) without skilled birth attendance. Maintaining clean delivery practices in home environments can be challenging for mothers and their birthing companions [2]. A recent analysis suggests that locally made kits linked with programmes to improve clean delivery practices are highly cost effective, at an estimated US$215 per life saved [9]. Kits usually include soap for washing the birth attendant's hands and mother's perineum, a plastic sheet to provide a clean delivery surface, a clean string for tying the umbilical cord, a new razor blade for cutting the cord, and pictorial instructions to illustrate the sequence of events during a delivery [7].

A recent systematic review on clean birth practices suggested that empirical evidence on the impact of clean delivery kits and clean delivery practices on neonatal mortality or sepsis-related neonatal deaths from community-based studies is surprisingly scarce [8]. A cluster-randomised controlled trial (cRCT) in rural Pakistan examined the effect on neonatal mortality of training traditional birth attendants (TBAs) and supplying them with clean delivery kits [10]. At the end of the study, neonatal mortality was 35 per 1,000 in the intervention clusters and 49 per 1,000 in control clusters (odds ratio [OR] 0.71, *p*<0.001). The specific contribution of kit use to the mortality reduction could not be estimated because the trial evaluated the impact of a broad antenatal care and delivery package. However, kits were used in 35% of deliveries in intervention clusters compared with only 3% in control clusters. Other studies included a cross-sectional survey from Egypt, which found an independent association between kit use and reduced cord infection (OR 0.42, *p* = 0.041), and a stepped-wedge randomised community trial in Tanzania in which cord infection was 12.6 times more likely (*p*<0.001) among neonates whose mothers did not use a kit [11],[12]. Four other studies of the effect of clean birth kits on cord infection summarised in a recent review had heterogeneous results [8]. In all, kits were included in larger integrated packages to improve neonatal and maternal outcomes. Other studies showed that, while kits modify practices directly linked to their physical components, for example use of a clean, boiled blade, they often do not affect more distal caring practices depicted in accompanying instructions and educational leaflets, for example early breastfeeding and wrapping the newborn infant [13]. Research evaluating the effectiveness of kits needs to take into account the effects of other interventions (e.g., concurrent kit promotion activities), as well as potential confounders that could influence their impact on neonatal mortality.

In this study we used data from the control arms of three cRCTs conducted by the authors among rural, underserved populations in South Asia, to explore associations between neonatal mortality, the use of clean delivery kits, and individual clean delivery practices. We had full access to individual participant data from these trials. Data from other previously conducted trials on clean delivery practices and kit use were not included as the heterogeneity of designs employed in other studies, which was noted in a recent systematic review, made it inadvisable to combine our estimates [8]. Our analysis had three objectives: first, to examine the association of kit use with neonatal mortality; second, to assess the association of neonatal mortality with individual clean delivery practices (hand washing, using a plastic sheet, use of gloves, sterilizing the blade, sterilizing the string, applying antiseptic to the umbilical stump, and dry cord care); third, to determine the cumulative effect on neonatal mortality of using four clean delivery practices, irrespective of kit use. The analyses were conducted for each site separately as well as using the pooled dataset for all sites, controlling for country of origin.

## Methods

### Ethical Approval

Ethical approval for the trials during which data for this study were collected came from the Institute of Child Health and Great Ormond Street Hospital for Children (UK) and the following in-country research ethics committees: the ethics committee of the Diabetic Association of Bangladesh (Perinatal Care Project, Bangladesh Diabetes Somity or BADAS); an independent ethics committee in Jamshedpur, India (Ekjut trial); and the Nepal Health Research Council. All trials were conducted in disadvantaged areas with high levels of female illiteracy. All participants gave consent in writing, by thumbprint, or verbally.

### Study Populations and Interventions

**Figure 1:**
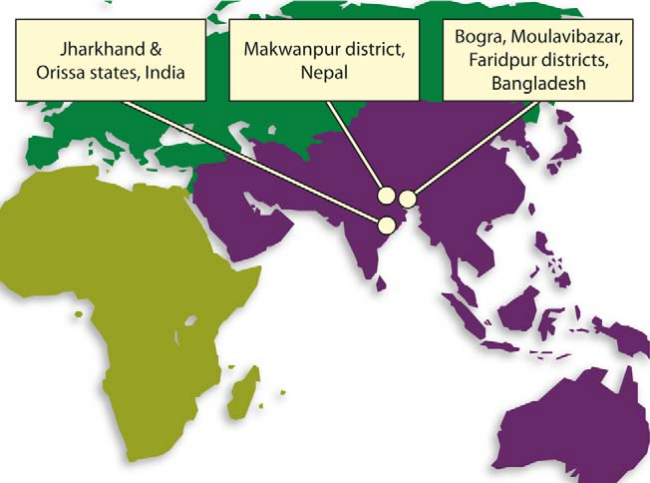
Location of study sites.

We used data from 19,754 home births available from the control arms of three community-based cRCTs carried out between 2000 and 2008 in India (*n* = 6,841), Bangladesh (*n* = 7,041), and Nepal (*n* = 5,872) [14]–[16]. Figure 1 shows their locations. Table 1 describes the characteristics of each study population, the timeline of studies, the contents of clean delivery kits available in each site, and baseline neonatal mortality rates. In Nepal, we used surveillance data from an additional six control clusters that were not part of the original cRCT. These clusters were located in the same district as the other clusters, were similar to them, and identical surveillance methods were used. In each of the cRCTs, clusters were randomised to intervention or control arms. Intervention clusters received a community-based participatory intervention within women's groups, aimed at improving maternal and newborn health. As these clusters received a complex intervention with the potential to confound or modify the association between kit use and clean delivery practices and mortality, we restricted our analysis to the control arms.

**Table 1:** Characteristics of the studies and populations included in the analysis.

Characteristics	India	Bangladesh	Nepal
Location	Three districts of Jharkhand and Orissa (eastern India): Keonjhar, West Singhbhum, and Saraikela	Three rural districts: Bogra, Maulvibazaar, and Faridpur	Makwanpur district
Study period	July 31, 2005 to July 30, 2008	Feb 1, 2005 to Dec 31, 2007	cRCT: Nov 1, 2001 to Oct 31, 2003. Intervention roll-out: 2003–2007
Study design	cRCT, open cohort.	Factorial design, cRCT, open cohort.	cRCT, matched design and closed cohort. Post cRCT, roll-out of intervention into control clusters.
Cluster characteristics	8–10 villages with residents classified as tribal or OBC.	Villages making up a union.	Village Development Committees.
*n* clusters analysed	18	5	18
Participants	Women aged between 15 and 49 y who had given birth in study period and their infants.	Women aged between 15 and 49 y who had given birth in study period and their infants.	Women aged between 15 and 49 y, married, and with potential to become pregnant in study period and their infants.
*n* births analysed	6,841	7,041	5,872
Neonatal mortality rate prior to intervention (per 1,000 live births)	58^a^	41^b^	60^b^
Contents of clean delivery kits	Soap, razor, plastic sheet, string, gauze. Instructions available in government kits only.	Soap, razor, plastic sheet, string, gauze. Instructions available in government kits only.	Soap, razor, plastic sheet, string, gauze. Plastic coin to use as surface to cut the cord. Instructions available in government kits only.
Individual clean delivery practices recorded separately from kit use	Hand washing, use of boiled blade to cut cord, type of cord care (dry or other), use of boiled thread to tie the cord, use of plastic sheet, and use of gloves.	Hand washing, use of boiled blade to cut cord, type of cord care (dry or other), use of boiled thread to tie the cord, use of plastic sheet, and use of gloves.	Hand washing, use of boiled blade to cut cord, type of cord care (dry or other).
Concurrent activities to promote clean delivery practices and kit use	In both intervention and control areas, strengthening the activities of village health and sanitation committees.	Training was provided to nurses, doctors, and paramedical staff in essential newborn care, including the six cleans.	Health service strengthening across intervention and control areas included training of all health workers on the six cleans.

a Neonatal mortality rate from cRCT baseline data.

b Neonatal mortality rate from district health survey data.

OBC, other backward class.

In all each study areas, kits were promoted and distributed through the health system as part of government initiatives to improve birth outcomes. In all sites, kits included the following as a minimum: soap, clean string, a razor blade, and a plastic sheet. Sterilisation of string and blade was recommended. In India, mothers received kits from health facilities, made some themselves, and also purchased some from each other as well as from TBAs. In Nepal, kits included a plastic disc against which the cord could be cut. Instructions on kit use were included in Nepal and Bangladesh, and in government manufactured kits in India. Data on kit use and individual clean delivery practices were collected in each of the studies. Our analysis was limited to live-born singleton infants delivered at home in control areas, for whom data on kit use were available.

### Surveillance Systems and Outcome Ascertainment

The sites had similar surveillance systems to monitor birth outcomes, and the same data collection procedures were followed in control clusters (included in this study) as in intervention clusters (excluded from this study) at all sites. Details of the individual surveillance systems can be found in previous publications [14]–[16],[17]. Briefly, in Nepal community-based monitors identified all pregnancies then followed up pregnant women to ascertain any births and deaths. In India and Bangladesh, one key informant per 250 households identified all births and reported birth outcomes and maternal deaths. Following an identification, an interviewer met with all mothers to verify the birth and/or death and administer a structured questionnaire to the mother, or, in case of a maternal death, to a relative. Following ICD 10, we defined a neonatal death as death to a newborn infant within the first 28 d of life [18]. All sites gathered information about the antenatal, delivery, and postnatal periods through a structured questionnaire administered to mothers in a non-blinded manner around 6 wk after delivery. In India and Bangladesh, interviewers asked about kit use and described its contents to mothers at the time of interview. In Nepal, interviewers showed a picture of a clean delivery kit to the respondent. If the respondent recognised it, they were asked if a kit had been used during delivery. Independent of mothers' knowledge and use of kits, information was collected on the following clean delivery practices: using a boiled instrument to cut the cord, hand washing, use of dry cord care, and antiseptic cord dressing. The WHO defines “dry cord care” as the practice of putting nothing on the newly cut umbilical cord, or cleaning soiled skin in the periumbilical area with soap and water, wiping it with a dry cotton swab or cloth, and allowing the area to air dry [19]. In our study sites, mothers were asked whether any substance was placed on their newborn's umbilical cord during their interview around 6 wk after delivery, and we coded their response as “dry cord care” if no substance had been applied. Information on the use of a boiled string to tie the cord, use of gloves and a plastic sheet was collected in Bangladesh and India, but not in Nepal.

### Data Collection and Management

Data were collected on paper, checked by auditors, entered by separate data entry operators, and cross-checked by data managers for data quality purposes. Databases were created in Microsoft Access or SQL Server. Separate datasets for each study and a pooled dataset consisting of information common to the three sites were then prepared for analysis in Stata, release 11.0 [20].

### Statistical Methods

We considered variables that might potentially confound or modify the association between kit use, clean delivery practices, and neonatal mortality on the basis of a priori knowledge. These confounders included; maternal age (years), education and reading ability, household assets, number of antenatal care visits, obstetric haemorrhage, preterm delivery, delivery assisted by a skilled birth attendant (doctor, nurse, or trained midwife), delivery assisted by a TBA, exclusive breastfeeding, fever in the 3 d preceding delivery, malpresentation, and season of birth. In site-specific analyses for Bangladesh and India, we adjusted for additional confounders including: cord wrapped around the infant's neck at birth, infant in poor condition at 5 min (poor or no cry, blue limbs, infant poorly active or no movement), maternal ability to independently access a health care facility, and parity. We compared differences in these potential confounders and effect modifiers between kit users and nonusers.

Neonatal and maternal characteristics and clean delivery practices were compared between respondents with complete and those with missing information on clean delivery kit use using chi-square statistics, to establish whether missing data could potentially bias subsequent analyses. As kit uptake was relatively low, data from three separate study sites were combined into a pooled dataset to increase the power to detect accurate estimates.

Analyses exploring the association of clean delivery kits with neonatal mortality were carried out using the pooled dataset and separately for the three sites. For each analysis, we examined the association of kit use with neonatal death using hierarchical logistic regression, controlling for all confounders common to the study sites to ensure comparability of results. Maternal age, parity, and number of antenatal care visits were treated as continuous variables. Two-way interaction terms were fitted between all potential confounders, kit use, and neonatal mortality where there was a plausible explanation.

We used similar methods for analyses of the association of clean delivery practices with neonatal mortality. First, we examined the individual association of each clean delivery practice with neonatal mortality in separate hierarchical logistic regression models, controlling for kit use and all other confounders. The Nepal dataset did not contain information on boiling the thread, use of a plastic sheet, or use of gloves, so these practices were evaluated using the pooled data from Bangladesh and India only, and separately for each of the two sites. Second, to determine if the four clean delivery practices documented in India and Bangladesh had an augmented collective benefit, we introduced into the model a covariate for the number of practices followed, along with kit use and potential confounders. A linear test for trend for number of clean delivery practices was applied to the hierarchical model, and a likelihood ratio statistic with *p*<0.05 considered significant. Antiseptic use was not included as limited incidence led to difficulties in model convergence.

We used data from 18, 18, and 5 population clusters in India, Nepal, and Bangladesh respectively, and we assumed that delivery practices would be more similar for births that occurred in the same cluster, than for births in other clusters. Likelihood ratio tests confirmed the clustered nature of the data on delivery practices in all three datasets (*p*<0.05), and we addressed it in the hierarchical models by using the Stata “xtmelogit” command, which provides maximum likelihood estimation using adaptive quadrature. There was no evidence of multicollinearity in any model.

## Results

### Study Population Characteristics

Univariable analyses revealed that kits were used for 18.4% (1,256) of home births in India, 18.4% (1,294), in Bangladesh, and 5.7% (335) in Nepal. The mean maternal age was 25.8, 24.7, and 27.2 y in India, Bangladesh, and Nepal, respectively. There was substantial variation in female literacy: in India, 76.4% (5,224) of mothers were illiterate, in Bangladesh 37.4% (2,634), and in Nepal 68.8% (3,896). In India, 4.9% (337) of home-delivered infants had a skilled birth attendant, compared with 1.1% (78) in Bangladesh and 0.4% (24) in Nepal.

Data on kit use were missing for 0.5% (38) of births in India and 2.1% (159) in Bangladesh. There were no missing data on kit use in Nepal because of the interview sequence described earlier. Because there were few missing data, we do not present differences between infants with missing data for kit use and those with complete data.

Table 2 presents a comparison of births with and without clean delivery kit use. Using a clean delivery kit was associated with neonatal survival in India and Bangladesh, but not in Nepal. Infants breastfed exclusively for the first 6 wk of life were more likely to have been delivered using a kit than nonexclusively breastfed infants in Bangladesh (*p*<0.001), but not in Nepal. Term infants were also more likely to have been delivered using a kit than preterm infants in India and Bangladesh (*p*<0.001), but not in Nepal. Kits did not necessarily guarantee clean delivery practices: in India, for example, hand washing with soap prior to delivery occurred in only 40% (480/1,256) of births at which a kit was used. Gaps in other clean delivery practices were found in all three sites for births at which a clean delivery kit was used, though in general clean delivery practices were more likely to be observed when a kit had been used.

**Table 2:** Comparison of deliveries with and without clean delivery kit use.

Factors Associated with Use of a Clean Delivery Kit	India (*n* = 6,841)		Bangladesh (*n* = 7,041)		Nepal (*n* = 5,872)	
	Used a Kit (*n* = 1,256)	Did Not Use a Kit (*n* = 5,585)	Used a Kit (*n* = 1,294)	Did Not Use a Kit (*n* = 5,747)	Used a Kit (*n* = 335)	Did Not Use a Kit (*n* = 5,537)
**Newborn health**						
Neonatal death, *n* (%)						
No	1,221 (97.2)	5,254 (94.1)*	1,267 (97.9)	5,550 (96.6)*	329 (98.2)	5,374 (97.1)
Yes	35 (2.8)	331 (5.9)	27 (2.1)	197 (3.4)	6 (1.8)	163 (2.9)
Baby exclusively breastfed, *n* (%)						
Yes	862 (68.6)	3,839 (68.8)	910 (70.3)	3,497(60.9)*	289 (86.8)	5,186 (94.4)*
No	394 (31.4)	1,745 (31.2)	384 (29.7)	2,248 (39.1)	44 (13.2)	307 (5.6)
Missing	0	1 (0.0)	0	2 (0.0)	2 (0.6)	44 (0.8)
**Clean delivery practices**						
Hand washing before assisting delivery, *n* (%)						
No	712 (59.7)	4,255 (80.2)*	72 (6.4)	1,482 (29.9)*	38 (12.5)	1,792 (48.8)*
Yes	480 (40.3)	1,054 (19.8)	1,056 (93.6)	3,478 (70.1)	267 (87.5)	1,878 (51.2)
Missing	64 (5.1)	276 (4.9)	166 (12.8)	787 (13.7)	30 (9.0)	1,876 (33.7)
Use of plastic sheet, *n* (%)						
No	775 (61.7)	5,520 (98.8)*	66 (5.1)	3,880 (67.5)*	na^a^	na
Yes	481 (38.3)	65 (1.2)	1,228 (94.9)	1,867 (32.5)	na	na
Use of boiled blade to cut cord, *n* (%)						
No	918 (77.9)	4,699 (87.0)*	288 (23.5)	2,101 (38.1)*	70 (21.1)	4,025 (73.2)*
Yes	260 (22.1)	699 (13.0)	938 (76.5)	3,408 (61.9)	262 (78.9)	1,475 (26.8)
Missing	78 (6.2)	187 (3.4)	68 (5.3)	238 (4.1)	3 (0.9)	37 (0.7)
Use of boiled thread to tie the cord, *n* (%)						
No	970 (80.5)	4,879 (89.8)*	306 (25.1)	2,417 (44.2)*	na	na
Yes	235 (19.5)	557 (10.2)	912 (74.9)	3,048 (55.8)	na	na
Missing	51 (4.1)	149 (2.7)	76 (5.9)	282 (4.9)	na	na
Use of gloves to assist delivery, *n* (%)						
No	1,041 (82.9)	5,513 (98.7)*	1,085 (83.8)	5,545 (96.5)*	na	na
Yes	214 (17.1)	72 (1.3)	209 (16.2)	202 (3.5)	na	na
Use of antiseptic to clean the cord, *n* (%)						
No	1,212 (96.5)	5,543 (99.2)*	1,223 (95.0)	5,509 (96.6)*	309 (95.1)	5,462 (99.8)*
Yes	44 (3.5)	42 (0.8)	64 (5.0)	192 (3.4)	16 (4.9)	12 (0.2)
Missing	0	0	7 (0.5)	46 (0.8)	10 (34.0)	63 (1.1)
Use of dry cord care practice, *n* (%)						
No	148 (11.8)	626 (11.2)	445 (34.6)	2,191 (38.4)*	109 (33.4)	1,332 (24.3)*
Yes	1,108 (88.2)	4,959 (88.8)	842 (65.4)	3,510 (61.6)	217 (66.6)	4,142 (75.7)
Missing	0	0	7 (0.5)	46 (0.8)	9 (2.7)	63 (1.1)
**Maternal characteristics**						
Maternal education, *n* (%)						
No education	818 (65.1)	4,312 (77.2)	359 (27.7)	2,002 (34.8)*	150 (45.7)	4,237 (79.4)
Primary	62 (4.9)	262 (4.7)	435 (33.6)	2,033 (35.4)	85 (25.9)	788 (14.7)
Secondary	376 (29.9)	1,011 (18.1)	500 (38.6)	1,712 (29.8)	93 (28.4)	314 (5.9)
Missing	0	0	0	0	7 (2.1)	198 (3.6)
Maternal reading ability, *n* (%)						
Unable to read	833 (66.3)	4,391 (78.6)*	632 (48.9)	2,339 (40.7)*	146 (44.5)	766 (14.4)*
Reads with difficulty	83 (6.6)	281 (5.0)	234 (18.1)	1,199 (20.9)	78 (23.8)	781 (14.6)
Reads with ease	340 (27.1)	913 (16.4)	426 (33.0)	2,204 (38.4)	104 (31.7)	3,792 (71.0)
Missing	0	0	2 (0.1)	5 (0.2)	7 (2.1)	198 (3.6)
Maternal age in years, *n* (%)						
<20	143 (12.0)	620 (12.0)*	237 (18.3)	903 (15.7)*	46 (13.7)	610 (11.0)*
20–29	766 (64.4)	3,131 (60.5)	822 (63.5)	3,671 (63.9)	225 (67.2)	3,249 (58.7)
30–39	269 (22.6)	1,355 (26.2)	224 (17.3)	1,098 (19.1)	57 (17.0)	1,381 (25.0)
40+	11 (0.9)	71 (1.4)	11 (0.9)	73 (1.3)	7 (2.1)	296 (5.3)
Missing	67 (5.3)	408 (7.3)	0	2 (0.0)	0	1 (0.0)
Caste or tribal group, *n* (%)						
Scheduled tribe^b^	880 (70.1)	4,190 (75.0)*	na	na	na	na
Scheduled caste^b^	53 (4.2)	214 (3.8)	na	na	na	na
Other backward class^b^	316 (25.2)	1,160 (20.8)	na	na	na	na
Household assets, *n* (%)						
All	230 (18.3)	922 (16.5)	561 (43.4)	1,807 (31.4)	159 (47.5)	1,094 (19.8)
Some	810 (64.5)	3,570 (63.9)	228 (17.6)	1,084 (18.9)	114 (34.0)	1,912 (34.5)
None	216 (17.2)	1,093 (19.6)	505 (39.0)	2,856 (49.7)	62 (18.5)	2,531 (45.7)
Parity, *n* (%)						
1	308 (24.5)	1,195 (21.4)*	483 (37.3)	1,765 (30.7)*	na	na
2	313 (24.9)	1,304 (23.3)	360 (27.8)	1,558 (27.1)	na	na
3	241 (19.2)	1,079 (19.3)	200 (15.5)	1,062 (18.5)	na	na
4	152 (12.1)	742 (13.3)	116 (9.0)	632 (11.0)	na	na
5	105 (8.4)	494 (8.9)	67 (5.2)	370 (6.4)	na	na
6	137 (10.9)	771 (13.8)	68 (5.2)	360 (6.3)	na	na
Mother can access a health facility independently, *n* (%)						
Always	125 (10.0)	661 (11.8)*	43 (3.3)	296 (5.1)*	na	na
Sometimes	376 (29.9)	1,470 (26.3)	328 (25.3)	2,026 (35.3)	na	na
Never without company	731 (58.2)	3,194 (57.2)	887 (68.6)	3,298 (57.4)	na	na
Never even with company	24 (1.9)	260 (4.7)	36 (2.8)	127 (2.2)	na	na
**Antenatal period**						
Number of antenatal care visits, *n* (%)						
0	263 (21.0)	1,765 (31.6)*	292 (22.6)	2,478 (43.1)*	51 (15.2)	3,389 (61.1)*
1	144 (11.5)	757 (13.6)	217 (16.8)	1,279 (22.3)	33 (9.9)	522 (9.4)
2	299 (23.9)	1,314 (23.5)	254 (19.7)	860 (15.0)	34 (10.1)	465 (8.4)
3	218 (17.4)	894 (16.0)	198 (15.3)	598 (10.4)	54 (16.1)	516 (9.3)
4	329 (26.2)	852 (15.3)	331 (25.6)	528 (9.2)	163 (48.7)	645 (11.7)
Missing	3 (0.2)	3 (0.1)	2 (0.2)	4 (0.1)	0	0
Bleeding during pregnancy, *n* (%)						
No	1,249 (99.4)	5,541 (99.2)	1,242 (95.6)	5,601 (97.5)*	320 (95.5)	5,375 (97.1)
Yes	7 (0.6)	44 (0.8)	52 (4.0)	145 (2.5)	15 (4.5)	162 (2.9)
Missing	0	3 (0.1)	0	0	0	0
**Delivery period**						
Preterm birth, *n* (%)						
Baby born at term	1,201 (95.6)	5,242 (93.9)*	1,268 (98.0)	5,521 (96.1)*	316 (94.3)	5,355 (96.7)*
Baby born after less than 9 mo gestation	55 (4.4)	343 (6.1)	26 (2.0)	226 (3.9)	19 (5.7)	182 (3.3)
Season of birth, *n* (%)						
Summer (March–June)	464(36.9)	1,902 (34.1)*	363 (28.1)	1,612 (28.1)	94 (28.1)	1,638 (29.6)
Rainy (July–October)	398 (31.7)	1,826 (32.7)	476 (36.8)	2,163 (37.6)	107 (31.9)	2,061 (37.2)
Winter (November–February)	394 (31.4)	1,857 (33.2)	455 (35.2)	1,972 (34.3)	134 (40.0)	1,838 (33.2)
Baby delivered by skilled delivery attendant, *n* (%)^c^						
Yes	171 (13.7)	166 (3.0)*	42 (3.2)	36 (0.6)*	14 (4.2)	10 (0.2)*
No	1,080 (86.3)	5,407 (97.0)	1,252 (96.8)	5711 (99.4)	321 (95.8)	5,527 (99.8)
Missing	5 (0.4)	12 (0.2)	0	0	0	0
Delivery by a TBA, *n* (%)						
Yes	475 (37.8)	2,135 (38.2)	186 (14.4)	1,693 (29.5)*	241 (72.4)	5,312 (96.7)*
No	781 (62.2)	3,450 (61.8)	1,108 (85.6)	4,054 (70.5)	92 (27.6)	181 (3.3)
Missing	0	0	0	0	2 (0.6)	44 (0.7)
Excessive bleeding during delivery, *n* (%)						
No	1,186 (94.4)	5,296 (94.9)	1,268 (98.0)	5,643 (98.2)	300 (89.6)	5,027 (90.8)
Yes	70 (5.6)	286 (5.1)	26 (2.0)	104 (1.8)	35 (10.4)	510 (9.2)
Missing	0	1 (0.0)	0	2 (0.0)	2 (0.6)	44 (0.8)
Malpresentation at birth						
No	1,239 (99.2)	5,508 (99.0)	1,265 (98.1)	5,611 (97.8)	334 (99.7)	5,468 (99.2)
Yes	10 (0.8)	55 (1.0)	24 (1.9)	126 (2.2)	1 (0.3)	42 (0.8)
Missing	7 (0.6)	22 (0.4)	5 (0.4)	10 (0.2)	0	27 (0.5)
Fever 3 d prior to delivery						
No	1,226 (97.6)	5,388 (96.5)*	1,274 (98.4)	5,617 (97.7)	303 (90.4)	4,776 (86.3)*
Yes	30 (2.4)	197 (3.5)	20 (1.6)	130 (2.3)	32 (9.6)	760 (13.7)
Missing	0	0	0	0	0	1 (0)
Infant appearance 5 min after delivery						
Normal	1,256 (100)	5,571 (99.9)	1,193 (94.2)	5,291 (93.2)	na	na
Asphyxiated	0 (0)	7 (0.1)	73 (5.8)	386 (6.8)	na	na
Missing	0	7 (0.1)	28 (2.2)	70 (91.2)	na	na
Umbilical cord wrapped around infant's neck at birth						
No	1,105 (88.0)	4,929 (88.3)	1,266 (97.8)	5,606 (97.6)	na	na
Yes	151 (12.0)	656 (11.7)	28 (2.2)	141 (2.5)	na	na

*Differences between clean delivery kit use and non-use tested using chi-square statistic and significant at *p*<0.05.

a Not applicable: data were not collected in the study.

b Standard terms used in Indian demographic surveys.

c Doctor, nurse, or trained midwife.

na, not available.

### Clean Delivery Kits, Clean Delivery Practices, and Risk of Neonatal Mortality

Table 3 presents results of analyses examining the association between kit use and neonatal mortality, within and across study sites. Kit use was associated with a 48% relative reduction in neonatal mortality in the pooled dataset (OR 0.52, 95% CI 0.39–0.68), and the association did not differ significantly between sites. Use of a kit was associated with a 57% relative reduction in neonatal mortality in India (OR 0.43, 95% CI 0.29–0.63), 32% in Bangladesh (OR 0.68, 95% 0.44–1.04), and 49% in Nepal (OR 0.51, 95% CI 0.17–1.51).

**Table 3:** Adjusted odds ratios for the association between clean delivery kit use and clean delivery practices with neonatal mortality.

Practices	All Countries	India (*n* = 6,841)	Bangladesh (*n* = 7,041)	Nepal (*n* = 5,872)
Use of a clean delivery kit^a^	0.52 (0.39–0.68)^b^	0.43 (0.29–0.63)	0.68 (0.44–1.04)	0.51 (0.17–1.51)
Use of a boiled blade to cut the umbilical cord^c^	0.73 (0.59–0.90)^b^	0.74 (0.51–1.08)	0.67 (0.49–0.92)	0.80 (0.48–1.33)
Washing hands prior to delivery^c^	0.89 (0.73–1.09)^b^	0.69 (0.51–0.94)	0.86 (0.61–1.20)	1.66 (1.06–2.65)
Use of dry cord care^c^	1.51 (1.21–1.88)^b^	1.34 (0.91–1.96)	3.29 (2.27–4.78)	0.48 (0.32–0.73)
Use of antiseptic to clean the cord only^c^	0.16 (0.04–0.64)^b^	0.31 (0.04–2.25)	0.12 (0.02–0.84)	na^d^
Use of boiled thread to tie the cord^e^	0.71 (0.56–0.90)^f^	0.60 (0.39–0.92)	0.77 (0.56–1.05)	na^g^
Use of plastic sheet^e^	0.69 (0.51–0.93)^f^	0.63 (0.31–1.26)	0.68 (0.47–0.97)	na^g^
Use of gloves^e^	0.65 (0.37–1.13)^f^	0.40 (0.16–1.00)	0.94 (0.46–1.91)	na^g^
Use of each additional clean delivery practice^e^	0.84 (0.77–0.92)^f^	0.77 (0.66–0.92)	0.89 (0.79–1.00)	na^g^

a Adjusted for clustering, maternal age, maternal education, maternal reading ability, household assets, bleeding in pregnancy, excessive bleeding during delivery, preterm delivery, exclusive breastfeeding for the first 6 wk of life, season, number of antenatal care visits, malpresentation at delivery, fever 3 d prior to delivery, and, for the pooled analysis, study site.

b Data available from India, Bangladesh, and Nepal, *n* = 19,754.

c Adjusted for the indicators above and the use of a clean delivery kit.

d It was not possible to obtain estimates for this model because of low numbers of cases where antiseptic was used; however, it was possible to include Nepal data in the pooled analysis.

e Adjusted for the indicators above, and for delivery by a TBA, cord wrapped around infant's neck at delivery, infant condition at 5 min, parity, delivery by a skilled birth attendant (doctor, nurse, trained midwife).

f Data available from India and Bangladesh, *n* = 13,882.

g Not applicable: data were not collected in the study.

Table 3 also describes the association of seven individual clean delivery practices with neonatal mortality for all sites combined and separately. The use of a boiled blade to cut the cord, antiseptic to clean the cord, a boiled thread to tie the cord, and a plastic sheet for a clean delivery surface were all associated with significant relative reductions in mortality when controlling for kit use and confounders common to all sites in the pooled dataset. Dry cord care was associated with significantly increased odds of death in the pooled dataset, as well as in India and Bangladesh. However, in Nepal, dry cord care was associated with significant relative reductions in neonatal mortality (OR 0.48, 95% CI 0.32–0.73).

Finally, Table 3 shows results for a pooled analysis combining data from all three countries to explore the association of between one and four clean delivery practices with neonatal mortality. With each additional clean delivery practice, we found a 16% relative reduction in mortality (OR 0.84, 0.77–0.92).

### Findings from Cause-of-Death Data

To check the plausibility of the effect sizes, we used cause-specific mortality data available from the control arms of the Indian cRCT to examine the association of kits with sepsis-related neonatal death, and with death due to the other two primary causes of newborn mortality (consequences of preterm birth and intrapartum-related deaths, or birth asphyxia). This analysis accounted for clustering, and used data drawn from 366 verbal autopsies analysed by physician review. Kit use was associated with strong relative reductions in sepsis-related mortality (OR 0.28, 95% CI 0.12–0.65), but also with relative reductions in mortality ascribed to prematurity and birth asphyxia (OR 0.51, 95% CI 0.35–0.76).

## Discussion

Results from our pooled analysis across study sites indicated a significant association between kit use and reduced mortality in rural South Asian communities. The non-significant results found in Nepal may be due to the small number of kit users in this sample, resulting in lack of power. The results also indicate the importance of individual clean delivery practices: a combination of hand washing, use of sterilised blade, use of boiled thread and plastic sheet was linearly associated with a reduction in neonatal deaths with each additional clean delivery practice used.

Many governments and nongovernmental organisations encourage the use of clean delivery kits, both with and without accompanying promotion programmes. Our study shows that distributing kits, even with instructions, does not guarantee that life-saving clean delivery practices will be used. These findings concur with those of a qualitative study from Nepal in which 51 mothers and TBAs were interviewed about their perceptions of clean delivery kits [21]. Few users took out the instructions for the kit, and when they did, they had difficulties understanding them. Delivery and postnatal practices—for example, cord care and immediate breastfeeding—are culturally patterned, and understanding the context in which kits are used is key to developing and evaluating culturally appropriate promotion activities [22].

Given the potential of kits to improve neonatal survival following home births, how can their use be promoted? Programmes have employed several approaches, including dissemination through health facilities, community health workers, and private providers such as pharmacists, but few of these initiatives have been evaluated. In our study sites, an intervention involving community mobilisation through participatory women's groups was used to improve birth outcomes. Women's groups discussed clean delivery and care-seeking behaviour through stories and games that facilitated discussions about prevention and care for typical problems in mothers and newborn infants. As a result of these discussions, some groups made and promoted clean delivery kits, resulting in significant increases in kit use within intervention clusters in Nepal and India [14],[15]. In a recent Pakistani trial, Lady Health Workers (LHWs) conducted participatory group sessions with mothers to promote beneficial practices in the antenatal, delivery, and postnatal period. Clean delivery kits were available from LHWs in both intervention and control clusters, but kit use for home deliveries was more common in the intervention clusters (35% versus 3%; *p*<0.0001) [23]. Findings from these trials suggest that group-based community interventions can significantly increase the use of clean delivery kits for home births.

The content and cost of kits also need consideration. Most kits do not currently contain antiseptic to clean the umbilical cord, and the WHO recommends dry cord care. In our study, dry cord care was associated with an increased likelihood of neonatal death in Bangladesh and India, but not in Nepal, a finding that needs to be interpreted with caution. A cRCT in Sarlahi district, Nepal, compared topical applications of chlorhexidine to the umbilical cord to dry cord care in reducing cord infections and neonatal mortality. Mortality was reduced by 34%, from 21.6 to 14.4 per 1,000, (OR 0.66, 95% CI 0.46–0.95) for those infants enrolled and treated within 24 h [24]. Other studies are underway.

At the time during which the trials included in this study took place, the cost of a clean delivery kit was US$0.44 in India (20 Indian rupees), US$0.40 in Nepal (30 Nepalese rupees), and US$0.27 in Bangladesh (20 Bangladesh taka). While the kit can be considered a low-cost intervention, there have been no studies on willingness to pay for kits, and these costs may still be prohibitive for the poorest women.

Our analysis was limited to home births. Initiatives to promote access to skilled care at birth in South Asia have already resulted in substantial increases in institutional deliveries [25],[26]. Since this trend is likely to continue in the future, further research is needed to understand the possible population-level impact on neonatal mortality of promoting kits through different channels, for example through women's groups, for community-based skilled birth attendants and in health facilities. In particular, we need to understand whether the promotion of clean delivery kits and clean delivery practices for home births dis-incentivises institutional deliveries, whether promoting kits for home births in the context of increasing institutional deliveries is cost-effective, and the potential of kits to prevent infections during institutional deliveries [8].

### Study Limitations

The associations found between kit use, other clean delivery practices, and neonatal mortality were greater than expected based on previous estimates of cause-specific neonatal mortality due to sepsis. We are circumspect about our findings, particularly in view of the possibility of residual confounding. It is likely that women who used kits and whose birth attendants adopted clean delivery practices were different from women who did not. For example, kit users may have performed other postnatal caring practices unaccounted for in our list of confounders, and these could have reduced the risk of neonatal death. Results from the analysis of cause-specific mortality data from India are encouraging in that they confirm the association of kit use with reduced sepsis deaths, but also puzzling in that they suggest that kit use was associated with reduced deaths from prematurity and birth asphyxia, albeit to a lesser extent. This result could be due to residual confounding, or a reflection of the limitation of verbal autopsies, and in particular of single-cause diagnoses; infection may further aggravate the consequences of prematurity and birth asphyxia. Recall bias is a further potential limitation, as women were not interviewed until about 6 wk after delivery. Recall bias following a neonatal death could lead to both under and over-reporting of kit use, and therefore to both over and under-estimation of the effect sizes seen in this study. There is also a possibility of social desirability bias, in that women may have reported desirable practice to interviewers. Over-reporting of kit use would tend to lead to an under-estimation of its true effect. Finally, women with missing data were significantly more likely to have experienced a neonatal death; excluding them from the analysis would also tend to reduce the observed magnitude of the effect.

### Conclusions

Our findings suggest that the use of clean delivery kits and clean delivery practices are associated with an increased likelihood of neonatal survival in rural settings where access to formal care and institutional deliveries are limited. The use of kits may not always be accompanied by clean delivery practices, and the latter should be emphasised when promoting them. Further research should explore the context of kit use in order to develop and test locally appropriate promotion strategies, as well as examine the potential of kits to improve neonatal survival in the context of increasing institutional delivery rates.

## Abbreviations

cRCT: cluster-randomised controlled trial
OR: odds ratio
TBA: traditional birth attendant
WHO: World Health Organization
